# Epigenetic regulation of MAGE family in human cancer progression-DNA methylation, histone modification, and non-coding RNAs

**DOI:** 10.1186/s13148-018-0550-8

**Published:** 2018-09-05

**Authors:** Yishui Lian, Lingjiao Meng, Pingan Ding, Meixiang Sang

**Affiliations:** 1 Research Center, the Fourth Affiliated Hospital of Hebei Medical University, Shijiazhuang, 050017 Hebei People’s Republic of China; 2 Tumor Research Institute, the Fourth Affiliated Hospital of Hebei Medical University, Shijiazhuang, 050017 Hebei People’s Republic of China

**Keywords:** MAGE, Epigenetics, Transcription regulator, E3 RING ubiquitin ligases

## Abstract

The melanoma antigen gene (MAGE) proteins are a group of highly conserved family members that contain a common MAGE homology domain. Type I MAGEs are relevant cancer-testis antigens (CTAs), and originally considered as attractive targets for cancer immunotherapy due to their typically high expression in tumor tissues but restricted expression in normal adult tissues. Here, we reviewed the recent discoveries and ideas that illustrate the biological functions of MAGE family in cancer progression. Furthermore, we also highlighted the current understanding of the epigenetic mechanism of MAGE family expression in human cancers.

## Background

The first member of the melanoma antigen gene (MAGE) was discovered in 1991, when Van der Bruggen et al. performed experiments to identify tumor antigens from melanoma cells [1]. The human MAGE family was divided into two categories in the light of their chromosomal location and expression pattern [2–4]. Nowadays, MAGE family was well known as tumor-associated antigens and comprises more than 60 genes, which share a conserved MAGE homology domain (MHD) [5]. Type I MAGEs are relevant cancer-testis antigens (CTAs) which contain MAGE-A, -B and -C subfamily members [6], and therefore are rarely expressed in normal adult tissues, but highly expressed in various cancers, including melanoma, breast cancer, prostate cancer, lung adenocarcinoma, esophageal squamous cell carcinoma, gastric cancer, bladder cancer, ovarian cancer, hepatocellular carcinoma, and brain cancer [7–16]. Type II MAGEs contain the MAGE-D, -E, -F, -G, -H, -L, and necdin genes, which are not limited to the X chromosome and are expressed in various tissues, such as brain, embryonic, and adult tissues [2–4].

MAGE family has specific functions in normal development and tumor progression. Type I MAGEs express normally only in testis or placenta, and their restricted expression suggests that they may function in germ cell development. Many studies have consistently showed that MAGE-A family may play an important role in spermatogenesis and embryonic development [4]. MAGE-B4 was found to be expressed during premeiotic germ cell differentiation, indicating that MAGE proteins may also play a role in developing oocytes [17]. MAGE-A proteins were detected by immunohistochemistry in the early development of the central nervous system (CNS) and the spinal cord and brainstem of peripheral nerves, revealing that MAGE-A protein was also involved in neuronal development [18]. Type II MAGEs are highly expressed in the brain and participate in various neural processes. These MAGE proteins might perform important functions in differentiation and neurodevelopment, thus their loss of function leads to a range of cognitive, behavioral, and developmental defects [4]. However, the biological functions and underlying regulatory mechanism of MAGE family expression in cancer is still not fully understood. The known tumor-related functions of MAGE family were summarized in Table 1.

**Table 1:** The known tumor-related functions of MAGE family

Type	Subtype	Gene name	Highly expressed tumor type	Biological functions
MAGE-I	MAGE-A	MAGE-A1	Melanoma; gastric cancer; endometrial cancer; esophageal squamous cell carcinoma; head and neck cancer	Activating p-C-JUN directly or through ERK-MAPK pathways; Repressing transcription by binding to SKIP and recruiting HDAC1
		MAGE-A2	Glioma; breast cancer	Degradation of P53, MDM2, MDM4; Increasing ER-dependent signaling
		MAGE-A3	Non-small-cell lung cancer; hepatocellular carcinoma	Degradation of P53, AMPKα1; Enhancing TRIM28-dependent degradation of FBP1
		MAGE-A4	Hepatocellular carcinoma; lung cancer	Inactivate the oncoprotein gankyrin
		MAGE-A5	Head and neck cancer; non-small-cell lung cancer	Not well characterized
		MAGE-A6	Breast, colon, and lung cancer	Degradation of P53, AMPKα1
		MAGE-A7	Non-small-cell lung cancer	Not well characterized
		MAGE-A8	Bladder cancer	Not well characterized
		MAGE-A9	Head and neck cancer; hepatocellular carcinoma; esophageal squamous cell carcinoma; breast, colorectal, lung, bladder cancer	Not well characterized
		MAGE-A10	Breast cancer; stomach cancer; melanoma; esophageal and head and neck squamous carcinoma; bladder, lung, hepatocellular carcinoma	Not well characterized
		MAGE-A11	Breast cancer; esophageal squamous cell carcinoma; head and neck cancer; non-small cell lung cancer; prostate cancer	Increasing Skp2-mediated degradation of cyclin A and p130; Decreasing Skp2-mediated degradation of E2F1 and Skp2 self-ubiquitination; Increasing the AR transcriptional activity
		MAGE-A12	Prostatic carcinoma and colorectal cancer; melanoma, bladder, lung, esophageal carcinoma; head and neck cancer	Promoting the ubiquitination of p21
	MAGE-B	MAGE-B1	Hepatocellular carcinoma	Not well characterized
		MAGE-B2	Hepatocellular carcinoma	Not well characterized
		MAGE-B3	Colorectal cancer	Not well characterized
		MAGE-B4–18	Not well characterized	Not well characterized
	MAGE-C	MAGE-C1	Cutaneous melanoma; breast, lung cancer	Not well characterized
		MAGE-C2	Hepatocellular carcinoma; breast, lung cancer; melanoma; gastrointestinal stromal tumors	Enhancing TRIM28-dependent degradation of FBP1; Inhibiting degradation of cyclinE; Increasing KAP1-Ser824 phosphorylation
		MAGE-C3–7	Not well characterized	Not well characterized
MAGE-II	MAGE-D	MAGE-D1	Breast cancer	Not well characterized
		MAGE-D2	Melanoma; gastric, colorectal cancer; hepatocellular carcinoma	Suppressing TRAIL-induced apoptosis
		MAGE-D3	Not well characterized	Not well characterized
		MAGE-D4	Glioma; hepatocellular carcinoma Colorectal, esophageal, lung cancer	Not well characterized
	MAGE-E	MAGE-E1	Glioma	Not well characterized
		MAGE-E2–3	Not well characterized	Not well characterized
	MAGE-F	MAGE-F1	Colorectal, ovarian, breast, cervical cancer; melanoma and leukemia	Not well characterized
	MAGE-G	MAGE-G1	Not well characterized	Not well characterized
	MAGE-H	MAGE-H1	Breast cancer; colorectal cancer	Upregulating mir-200a/b expression via association with p73
	MAGE-L2	MAGE-L2	Not well characterized	Not well characterized
	NECDIN	NECDIN	Melanoma, prostate and breast cancer; leukemia; urothelial carcinoma	Repression in a STAT3-dependent manner

In this review, we summarized these exciting advances and discoveries concerning the biological functions of MAGE family in cancer progression. We also take a comprehensive look at the current understanding of the epigenetic mechanism of MAGE family expression in human cancers. This provides an outlook on cancer therapeutic approaches that target MAGE family.

## Biological functions of MAGEs in cancer progression

### MAGEs function as regulators of E3 RING ubiquitin ligases

Some studies have explored the function of MAGE proteins in cancer cells, and they were observed to promote cancer cell survival, tumor formation, and metastasis [19, 20]. Members of all type I MAGE protein families promoted tumor cell viability and inhibited cell apoptosis, therefore providing a growth advantage to melanoma and other malignancies [21]. MAGE-A3 and A6 were critical for cancer cell survival, and MAGE-A3/6-TRIM28 E3 ubiquitin ligase complex was found to degrade AMPKα1 resulting in downregulating AMPK signaling during tumorigenesis [22].

**Fig. 1:**
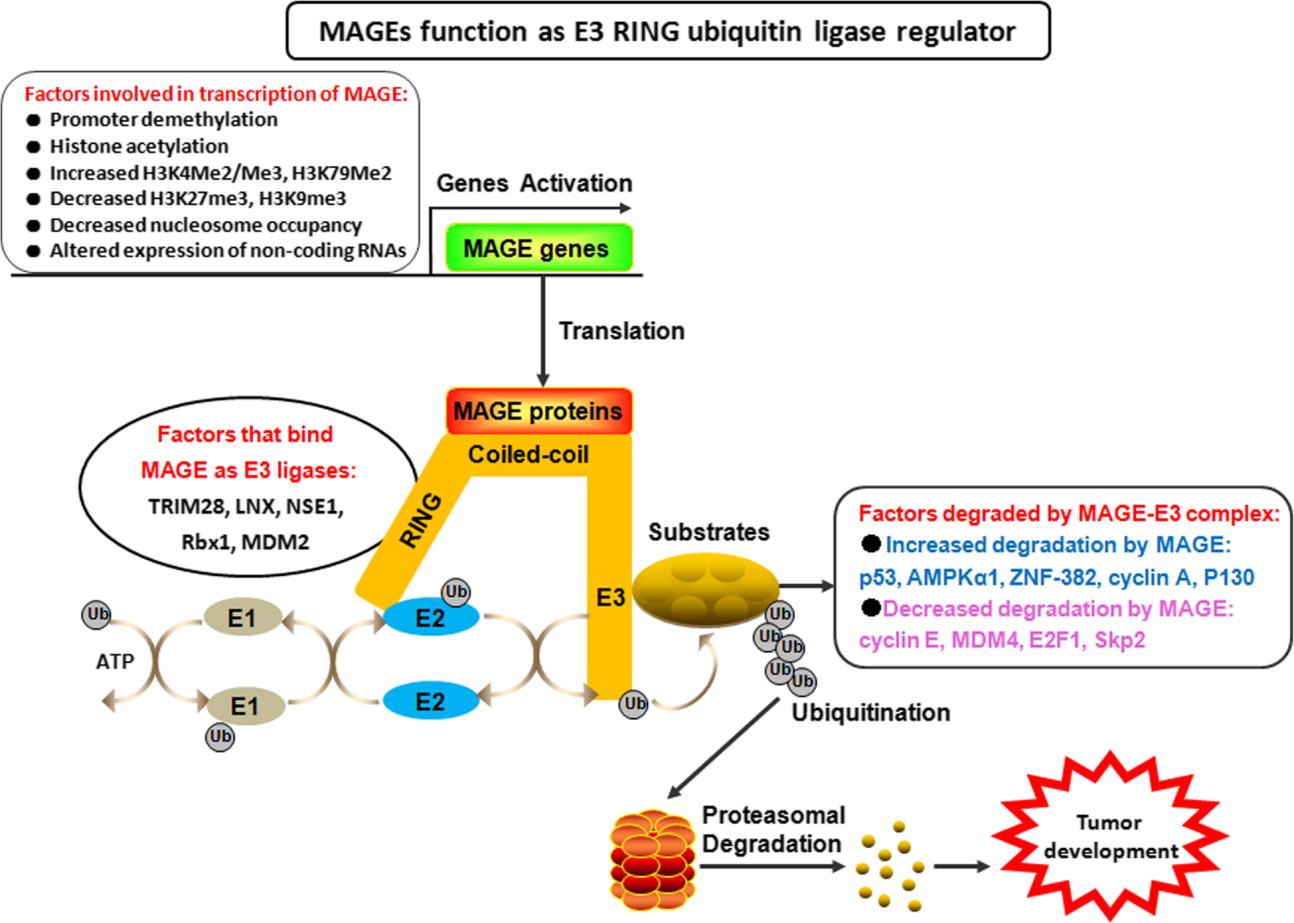
MAGEs function as regulator of E3 RING ubiquitin ligases. MAGE genes were activated by some epigenetic regulation factors such as DNA demethylation, histone acetylation, decreased nucleosome occupancy, and altered expression of non-coding RNAs. Then they were translated to proteins which could bind directly to RING domain proteins and act as scaffold of RING domain proteins to their substrates, thus regulating (increase or decrease) the ubiquitin ligase activity of RING domain proteins, which plays an important role in tumor development.

Recently, multiple MAGE proteins were found to form complexes with RING domain proteins, such as MAGE-A2/C2-TRIM28, MAGE-B18-LNX, MAGE-G1-NSE1 complexes, etc. [23, 24]. RING domain is a cysteine-rich domain that normally forms a cross-brace structure that typically coordinates two zinc ions [25]. RING domain proteins are proved to be a big E3 ubiquitin ligase family, which bind to and localize E2 ubiquitin-conjugating enzymes to substrates for ubiquitylation [26, 27]. MAGE proteins bind directly to RING domain proteins and act as scaffold of RING domain proteins to their substrates, thus regulating the ubiquitin ligase activity of RING domain proteins (Fig. 1). In particular, MAGE-A2, -A3, -A6, and -C2 were found to bind TRIM28, also known as KAP1, TIF-1beta or Krip125, therefore inducing the degradation of tumor suppressor p53 [23]. Recently, Potts and their colleagues reported MAGE-A3/6-TRIM28 complex ubiquitinates and degrades the tumor suppressor AMPKα1, thus leading to the inhibition of autophagy and activation of mTOR signaling [22, 28]. MAGE I binding to KAP1 induced the poly-ubiquitination and degradation of the substrate ZNF382 [29]. ZNF382 is one of KRAB domain zinc finger transcription factors (KZNFs) family member, which is involved in cell apoptosis and tumor suppression [30]. KZNFs bind the KAP1 protein and direct KAP1 to specific DNA sequences where it suppresses gene expression by inducing localized herterochromatin characterized by histone 3 lysine 9 trimethylation (H3K9me3). The binding of MAGE to KAP1 induces the degradation of ZNF382 leading to the decreased KAP1 binding to ID1 and the increased expression of oncogene ID1 [29]. Thus, it appears that cancer-specific up-regulation of MAGE family triggers ubiquitination and degradation of multiple tumor suppressors, such as p53, AMPKα1, and ZNF382 through binding to RING domain protein KAP1, promoting tumorigenesis and aggressive growth. Therefore, identification of novel small molecules that inhibit protein–protein interactions between MAGE and KAP1 may be a potential therapeutic strategy for cancer-bearing MAGE expression [31].

RING-box protein 1 (Rbx1), another RING domain containing protein, is a RING component of the largest E3 ligases SCF complex [32]. SCF complex consists of Rbx1, Cullin1, Skp1, and F-box protein family, and degradation of SCF-dependent proteolysis can cause a variety of diseases including cancer [32–34]. MAGE-C2 binds directly to Rbx1 and inhibits ubiquitin-dependent degradation of cyclin E, and promotes melanoma cell cycle progression at G1-S transition [35]. In addition, MAGE-A2 was reported to associate with MDM2, a RING finger-type E3 ligase that mediates ubiquitylation of more than 20 substrates including mainly p53, MDM2 itself, and MDM4. And the interaction of MAGE-A2 with MDM2 inhibits the E3 ubiquitin ligase activity of MDM2, thus increasing the level of MDM4. However, it does not affect p53 turnover mediated by MDM2 [36]. MAGE-A11 interacts with Skp2, the substrate recognition protein of the Skp1-Cullin1-F-box E3 ubiquitin ligase, and increases Skp2-mediated degradation of cyclin A and p130, but decreases Skp2-mediated degradation of E2F1 and Skp2 self-ubiquitination by sequestering and inactivating Skp2 via forming an E2F1-MAGE-A11-Skp2 complex [37].

### MAGEs function as transcriptional regulators

**Fig. 2:**
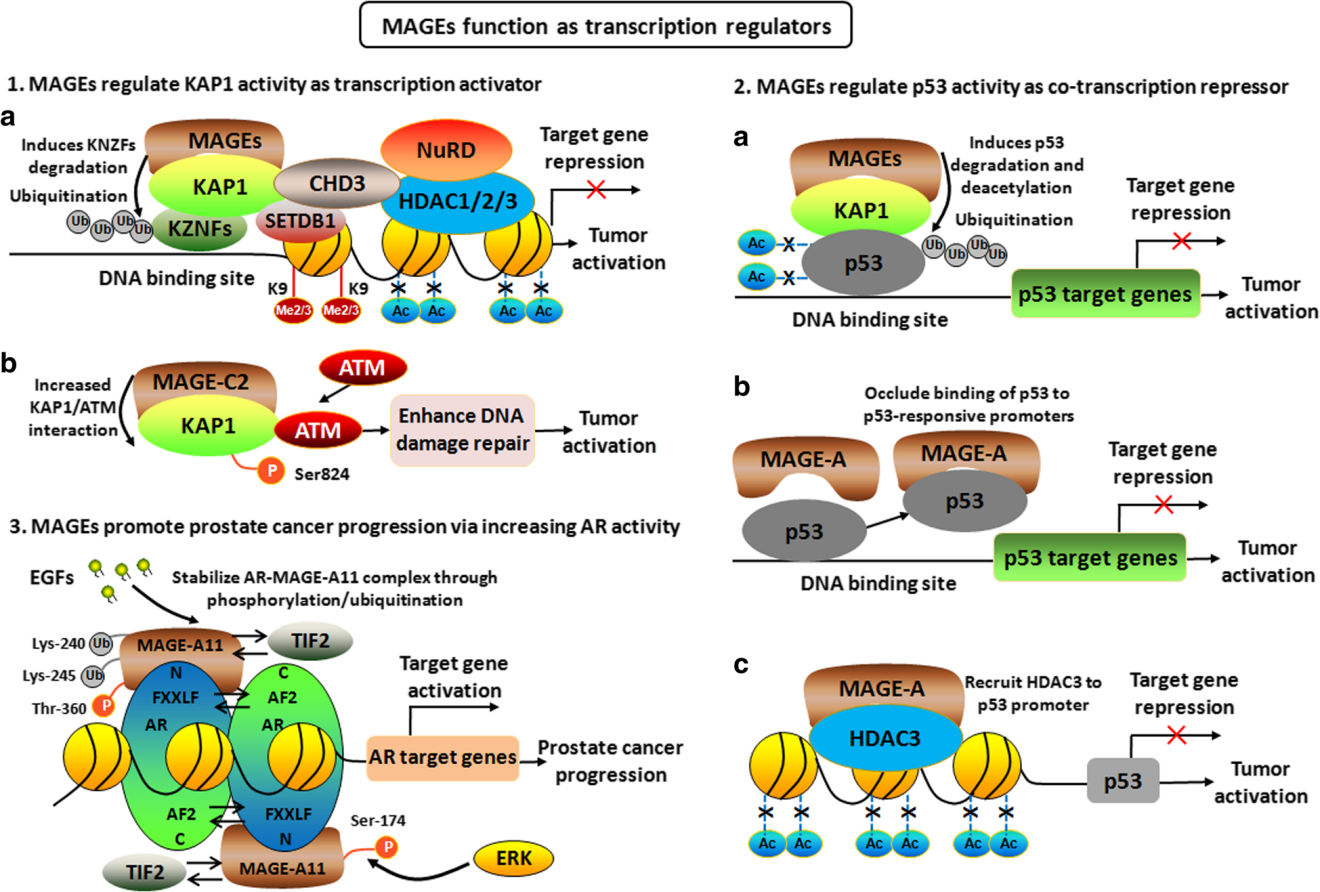
MAGEs function as transcription regulators. (**1**). MAGEs regulate KAP1 activity as transcription activator. a KAP1 functions as a molecular scaffold for gene-specific silencing by targeting of specific promoters through the KRAB protein zinc finger motifs, promotion of histone deacetylation via the NuRD/histone deacetylase complex, histone H3-K9 methylation via SETDB1 and recruitment of HP1 protein. b MAGE-C2 binds KAP1 and increases ATM-induced phosphorylation of KAP1-Serine 824 (Ser824), thus enhancing DNA damage repair and tumor activation. (**2**). a MAGEs binding to KAP1 induces p53 degradation and repression of p53 targeted genes. b MAGE-A proteins can directly interact with p53 leading to obstruction of p53 binding to p53-responsive promoters. c MAGE-A proteins also inhibit p53 transcription functions by recruiting HDAC3 to the binding sites of p53 promoter. (**3**). MAGEs promote prostate cancer progression via increasing AR activity. MAGE-A11 binds NH2-terminal FXXLF motif of AR and increases AR transcriptional activity by modulating AR interdomain interaction. EGFs stabilize AR-MAGE-A11 complex through the site-specific phosphorylation of Thr-360 and subsequent ubiquitinylation of Lys-240, Lys-245 within MAGE homology domain.

The binding of MAGE-C2 to KAP1 increases the interaction between KAP1 and ATM, and increased KAP1-Ser824 phosphorylation. Therefore, MAGE-C2 may promote tumor growth by phosphorylation of KAP1-Ser824 and the enhancement of DNA damage repair [38]. KAP1 seems to function as a molecular scaffold that coordinates at least four activities necessary for gene-specific silencing, including (a) targeting of specific promoters through the KRAB protein zinc finger motifs; (b) promotion of histone deacetylation via the NuRD/histone deacetylase complex; (c) histone H3-K9 methylation via SETDB1; and (d) recruitment of HP1 protein [39]. KAP1 also regulates DNA repair through the phosphorylation of KAP1-Serine 824 (Ser824) by ataxia-telangiectasia-mutated (ATM) kinase [40] (Fig. 2-1). As a scaffolding protein, KAP1 interacts with p53 and acts as a co-repressor of p53 expression and function. MAGE suppression decreases KAP1 complexing with p53, increases acetylation of p53, and activates p53 responsive reporter genes. Class I MAGE protein may promote tumor development at least in part through inhibiting p53 activation [21]. In addition, MAGE-A proteins can directly interact with p53. This direct interaction may occlude the binding of p53 to p53-responsive promoters, lead to the decreased p53-dependent transcription, cell cycle arrest, and apoptosis [41]. In multiple myeloma, the interaction of MAGE-A proteins with p53 was shown to inhibit apoptosis through repression of bax and stabilization of survivin [42]. MAGE-A proteins also inhibit p53 transcription functions, at least in part by recruiting HDAC3 to the binding sites of p53 promoter, leading to resistance to anti-tumor agents [43]. MAGE-A1 was reported to repress transcription through binding to ski interacting protein (SKIP), a transcription regulator, and recruiting HDAC1 [44]. Through forming complex with p53 and estrogen receptor α (ERα), MAGE-A2 represses p53 pathway and increases ER-dependent signaling, therefore contributing to tamoxifen-resistance of ER-positive breast cancer [45] (Fig. 2-2).

**Fig. 3:**
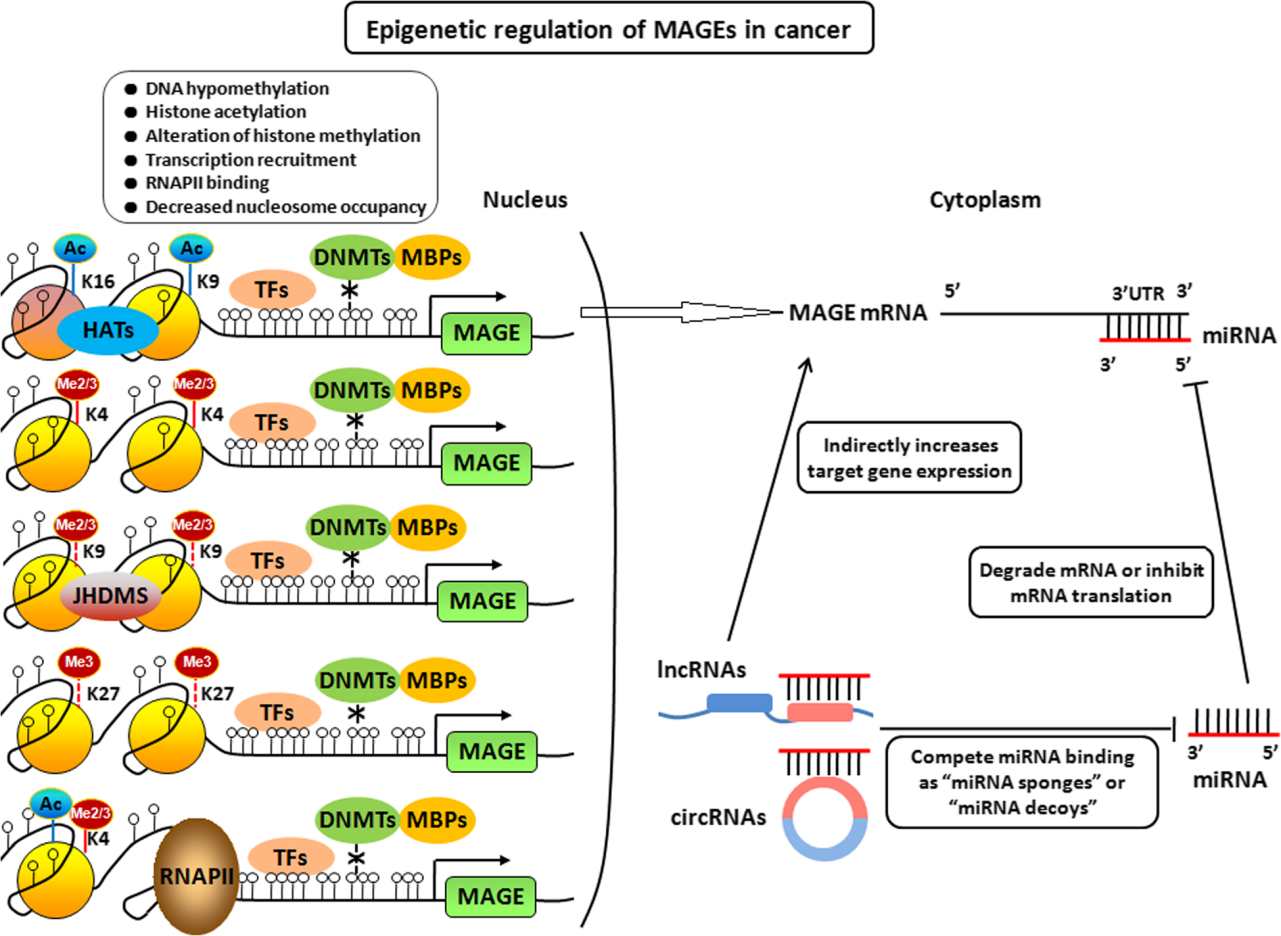
Model of epigenetic regulation of MAGEs in cancer progression. MAGE family can be activated by DNA hypomethylation, histone acetylation, histone methylation, and nucleosome depletion, eventually contributing to oncogenesis. At the same time, MAGEs might be regulated by ceRNA network through miRNAs as the mediators.

MAGE-A11 was found to play a crucial role in the androgen receptor (AR) signaling network in prostate cancer. MAGE-A11 forms a complex with AR by binding NH2-terminal FXXLF motif of AR and increases the AR transcriptional activity by modulating AR interdomain interaction [46, 47]. The increased expression of MAGE-11 facilitates prostate cancer progression by enhancing AR-dependent tumor growth [48]. Epidermalgrowth factor (EGF) stabilizes the AR-MAGE-A11 complex and increases androgen-dependent AR transcription activity through the site-specific phosphorylation of Thr-360, and subsequent ubiquitinylation of Lys-240 and Lys-245 within MAGE homology domain [49]. Further studies showed that the interaction between AR and MAGE-A11 is mediated by AR NH2-terminal FXXLF motif binding to a highly conserved MAGE-A11 F-box (residues 329-369) in the MAGE homology domain, and that the interaction is modulated by serum stimulation of mitogen-activated protein kinase phosphorylation of MAGE-A11 Ser-174 [50] (Fig. 2-3). In addition, MAGE-A11 also functions as a transcriptional coregulator through interacting with progesterone receptor (PR), steroid receptor-associated p300 and p160 coactivators, and contributes to cell cycle progression through interacting with p107 and E2F1 transcription factors which are important for cell cycle progression and induction of apoptosis [51, 52].

## Epigenetic regulation of MAGE family in cancer

As a CTA subfamily, type I MAGE-A gene expression is restricted to cancer cells and testis. However, the precise regulatory mechanisms of MAGE family expression are still not fully understood. Epigenetic regulation seems to play an important role in MAGE expression.

### DNA methylation plays critical role in the regulation of MAGEs expression

Contrary to the high homology of MAGE genes, their promoters are less homologous. These promoter regions contain some binding sites for the transcription factors. The hypermethylation of these sites may be involved in the silence of MAGE genes. For example, the promoter of MAGE-A1 gene contains binding sites for the transcription factors Ets and Sp1, whereby the Ets proteins are responsible for the high transcriptional activation. The hypermethylation of CpG dinucleotides on the MAGE-A promoters may prevent the binding of these activators to their motif, and consequently inhibiting the promoter activity [53, 54]. MAGE-A1 promoter was reported to be highly methylated in somatic tissues. In contrast, it is largely unmethylated in male germ cells and in tumor cells that express this gene [55]. Moreover, the expression of MAGE-A1 can be induced by the demethylating agents in cells that do not express this gene. These observations suggested that DNA methylation is an essential component of MAGE-A1 repression in somatic cells. MAGE-A11 expression is increased during prostate cancer progression and castration-recurrent growth of prostate cancer, which is resulted from the hypomethylation of CpG sites directly proximal to the MAGE-A11 transcription start site (TSS) [54]. MAGE-A11 expression is also correlated with DNA hypomethylation at its TSS in epithelial ovarian cancer, which is associated with the global DNA hypomethylation [54]. The demethylating agent decitabine was able to reduce MAGE-A11 promoter methylation levels. Its promoter activity is partially dependent on the transcription factor Sp1. Sp1 inhibitor mithramycin A (MitA) could cause a dose-dependent reduction in MAGE-A11 promoter activity and endogenous MAGE-A11 expression. In addition, DNA demethylating agent-mediated MAGE-A11 induction could be inhibited by MitA treatment [56]. Taken together, DNA methylation plays a primary role in MAGE-A11 gene silencing.

In mammalian, DNA methylation is regulated by two DNA methyltransferases (DNMTs) families: the so-called “de novo” methyltransferase of DNMT3 family and the “maintenance” methyltransferase DNMT1 [57]. In colon cancer cells, genetic knockout of DNMT1 caused moderate activation of X-link cancer/germline (CG-X) genes including MAGE-A1, NY-ESO-1, and XAGE-1, and DNMT3b knockout had a negligible effect on CG-X gene activation. However, double knockout of DNMT1 and DNMT3b caused dramatic hypomethylation of promoters and robust induction of these CG-X genes [58]. Similarly, in MZ2-MEL cells, down-regulation of DNMT1, but not DNMT3A and DNMT3B, induced the activation of MAGE-A1 gene, suggesting that DNMT1 has a predominant role for methylation maintenance of MAGE-A1 [59]. Therefore, both DNMT1 and DNMT3 family, participate in, and are necessary for, effective CpG island hypermethylation of MAGE genes.

Some methyl-CpG-binding domain (MBD) proteins, which are able to bind methylated DNA, have been reported to contribute to the silencing of MAGE-A genes as modulator [60]. Most hypermethylated promoters are occupied by MBD proteins, whereas unmethylated promoters generally lack MBDs. Treatment of demethylating agents causes hypomethylation of CpG islands, MBD release, and gene re-activation, reinforcing the notion that the association of MBDs with methylated promoters is methylation-dependent [61]. In all MBD-containing proteins, MBD1 differs from other members due to its unique structure and specific function in gene regulation. Except for the conserved MBD domain at its N-terminal, it also has a transcriptional repression domain (TRD) at its C-terminal [62]. These two domains are related to the interaction between MBD1 and other proteins. However, the MBD domain mediates the binding of MBD1 to the methylated DNA, but the TRD domain regulates the transcriptional repression of target genes. In addition, MBD1 has two or three specific CXXC domains distinct from other MBD-containing proteins. The number of CXXC motifs varies among different MBD1 isoforms and depends on whether MBD1 binds to the unmethylated DNA. The first two CXXC domains (CXXC1 and CXXC2) allow MBD1 to bind to the methylated DNA, but the presence of the third CXXC domain (CXXC3) enables MBD1 to bind to DNA irrespective of its methylation status [63]. MBD1 binds to methylated as well as unmethylated MAGE-A gene promoters, and leads to the repression of the promoters. Repression of unmethylated genes depends on the third CXXC domain, and repression of methylated genes requires the MBD domain. MBD1mut, which lacks the MBD domain and harbors a non-functional TRD, showed no effect on MAGE-A gene expression [60]. The isoform MBD1v1 which contains the additional third CXXC domain could repress MAGE-A gene promoters regardless of their methylation status. However, although MBD1v3 lacks the third CXXC domain, it also has a weak repression on the unmethylated MAGE-A gene promoters, suggesting that the two other CXXC domains may also contribute to the repression of unmethylated MAGE-A promoters, however, with a weaker affinity [60]. These two kinds of binding to both methylated and unmethylated DNA enable MBD1 to act in different epigenetic regulations for MAGE-A genes. Another methyl-CpG binding protein, MeCP2, was also found to regulate MAGE-A11 expression in ESCC progression [64].

MAGE-A gene promoters contain Ets motifs, and the transcription factor Ets has been shown to be responsible for the high transcriptional activation of MAGE-A1 [65]. Ets-1 over-expression could result in the activation of MAGE-A promoters. However, the trans-activator Ets-1 could not abrogate the MBD-1 mediated suppression, suggesting that binding of MBD1 to the unmethylated MAGE-A gene promoter lead to gene repression which could not be abrogated by Ets-1 [60]. MAGE-A11 was also reported to be stimulated via DNA demethylation, histone acetylation and histone methylation, resulting in strengthened ESCC proliferation [64]. These data revealed why promoter demethylation results in the activation of MAGE-A genes. In general, DNA methylation is dominant over other epigenetic mechanisms for CTA (including MAGE) gene repression [66].

### Post-translational modifications of histone play accessory roles in the regulation of MAGE expression

DNA methylation is intertwined with the post translational modification of histone [67]. Although hypermethylation of CpG-rich MAGE-A promoters plays a crucial role in the silencing of MAGE-A genes, several studies have shown that up-regulation of MAGE-A genes could not be always observed, although tumor cells were treated by the DNA methylase inhibitor DAC. Histone deacetylases inhibitor trichostatin A (TSA) was able to significantly up-regulate DAC-induced MAGE-A gene transcription, although treatment of several tumor cells with TSA alone had only small influence on MAGE-A gene expression, suggesting that histone deacetylation, which leads to a compact and transcriptionally inactive chromatin structure, also contributes to the repression of MAGE genes [58, 68]. The increased abundance of Ac-H3K9 at MAGE-A gene promoters correlates with increased MAGE-A gene expression. In addition, dual DNMT1/DNMT3b knockout resulted in large increases of AC-H3K9 level at MAGE-A promoters, which correlated with increased MAGE-A expression in cells [58]. Fibroblast growth factor receptor 2-IIIb (FGFR2-IIIb) could suppress MAGE-A3/A6 gene expression through increasing histone deacetylation and histone methylation in thyroid cancer [69]. These studies suggested that not only DNA hypermethylation but also histone deacetylation is responsible for the mechanism underlying MAGE-A gene silencing. Histone deacetylation could lead to a compact and transcriptional inactive chromatin structure, which is involved in the partial repression of MAGE-A genes in tumor cells and may impede their activation. However, in the DNA hypermethylated cells, MAGE-A genes could not be induced by TSA, suggesting that DNA methylation plays a primary role in MAGE-A gene repression, and histone deacetylation plays an accessory role in cells with hypermethylated MAGE-A genes (Fig. 3).

Histone lysine methylation also affects MAGE genes expression in cancer cells [70]. The increased level of H3K9me2 at MAGE-A promoters correlates with a lack of gene expression, whereas an increased abundance of H3K4me2 at these promoters correlates with increased MAGE-A gene expression [59]. MAGE-A high-expressed tumor cells exhibited increased occupancy of RNA polymerase II, enrichment of euchromatin/activation marks such as H3K4Me2, H3K4Me3, H3K79Me2, total H3Ac, H3K9Ac, toal H4Ac, and H4K16Ac, with decreased occupancy of SirT1 as well as polycomb repressor complex (PRC-2) components (KMT6, EED, and SUZ12), and associated PRC-2 mediated repression mark, H3K27me3 [64]. Knockdown of LSD-1 (KDM1) and JARID1B (KDM5B) that mediate demethylation of mono-, di-, and trimethylated H3K4, or the histone lysine methytransferase KMT6 that mediateds trimethylation of H3K27 significantly enhanced DAC-mediated activation of MAGE-A genes in lung cancer cells [70]. DZNep, as an EZH2 inhibitor, could decrease KMT6 and H3K27me3 levels within MAGE-A promoters, and significantly enhanced DAC-mediated induction of MAGE-A genes (Fig. 3).

### Nucleosome occupancy in the regulation of MAGE expression

Nucleosomes are the basic structural units of eukaryotic chromatin [71]. Increasing evidences revealed that nucleosomes and their position, in concert with other epigenetic mechanisms, such as DNA methylation, histone modifications, changes in histone variants, as well as small noncoding regulatory RNAs, play essential roles in the control of gene expression [72]. Most importantly, nucleosomes are depleted at promoter, enhancer, and terminator regions, which allow the access of transcription factors and other regulatory proteins [73, 74]. Nucleosome occupancy and positioning are dynamic processes during development as well as in response to different environmental conditions. Therefore, nucleosome positioning and occupancy are determined by combined action of DNA sequence features, transcription factors, chromatin remodelers, and histone modifications [75]. Nucleosome positioning can direct DNA methylation patterns, whereas DNA methylation also can dictate nucleosome occupancy at numerous genomic loci in human cancer cells [76]. For the epigenetic regulation of MAGE-A11, DNA methylation regulates nucleosome occupancy specifically at the − 1 positioned nucleosome of MAGEA11. Methylation of a single Ets site near the transcriptional start site correlated with − 1 nucleosome occupancy and, by itself, strongly repressed MAGEA11 promoter activity. Thus, DNA methylation regulates nucleosome occupancy at MAGEA11, and this appears to function cooperatively with sequence-specific transcription factors to regulate *MAGE-A11* gene expression [56].

### Crosstalk between DNA methylation, histone modifications, and nucleosome occupancy

In addition to performing their individual roles, DNA methylation, histone modifications, and nucleosome occupancy work together at multiple levels to determine gene expression status [77]. The crosstalk between DNA methylation and histone modifications can occur in two ways. Firstly, DNA methylation established can lead to the recruitment of MBPs and other transcription regulatory proteins. These proteins can recruit the “writers” of histone modifications followed by the recruitment of “readers” and/or “erasers”. Secondly, histone modifications can directly or indirectly recruit the methyl writers (such as DNMTs) to establish DNA methylation. Furthermore, DNA methylation and nucleosome positioning appear to be linked with transcription factor binding and gene expression in a complex manner [78, 79]. The crosstalk between DNA methylation, histone modifications, and nucleosome occupancy further enhance the complexity of epigenetic regulation of MAGE gene expression, which determines and maintains their function in cancer cells (Fig. 3).

### Non-coding RNAs including microRNAs (miRNAs) and competing endogenous RNA (ceRNA) regulate MAGEs expression in cancer progression

It has been demonstrated that approximately 5–10% of the sequence is transcribed in human genome. Among transcripts, about 10–20% are the protein-coding RNAs, and the rest 80–90% are non-protein-coding RNAs (ncRNAs). MAGE family was also regulated by ncRNAs. MiRNAs, a novel class of gene regulator, are a class of small non-coding RNAs of ∼ 22 nucleotides in length that regulate gene expression through post-transcriptional silencing of target genes [80]. Sequence-specific base pairing of miRNAs with 3′ untranslated region (3′UTR) of target mRNA within the RNA-induced silencing complex results in the degradation or translational inhibition of target mRNAs [81]. There also exist a lot of miRNAs-binding sites at the 3′UTR of MAGE gene mRNAs. MiR-34a was reported to directly bind the 3′UTR of several MAGE-A mRNAs including MAGE-A2, -A3, -A6, and -A12, and thus inhibiting the expression of MAGE-A members [82]. In addition, miR-874 could directly bind the 3′UTR of MAGE-C2 and at least in part negatively regulate the expression of MAGE-C2 in cancer cells [83]. In addition, miRNAs can also modulate epigenetic regulatory mechanisms in cells by targeting enzymes responsible for DNA methylation or histone modifications, which potentially could indirectly influence MAGE expressions [84, 85].

For many years, it is believed that miRNAs regulate gene expression in a simple “miRNA→mRNA→protein” pattern. However, in recent years, it has been found that some RNAs contain the same conservative miRNA binding sites and reduce miRNA availability for its mRNA targets by competing for miRNA binding as “miRNA sponges” or “miRNA decoys” [86, 87]. Based on these finding, the competing endogenous RNA hypothesis was proposed [88]. According to the ceRNA hypothesis, the role of miRNAs in regulating gene expression has thus been amended from that of an “initiator” to a “mediator,” and the regulation pattern has been amended from “miRNA→mRNA” to network-based “ceRNAs→miRNAs→mRNAs” [89]. Long non-coding RNAs (lncRNAs), circular RNAs (circRNAs), mRNAs and pseudogene transcripts are all revealed to act as ceRNAs and regulate the target genes by competing for the same miRNAs in the available miRNA pools [90–93]. In our recent study, MAGE-A family was found to be regulated by the circRNA-miRNA-mRNA axis in ESCC progression [94]. Taken together, MAGE family might be regulated by ceRNA network through miRNAs as the mediators (Fig. 3).

## Conclusion

MAGEs are expressed in a variety of human cancers, and drive tumor progression through various mechanisms, which eventually results in the tumor growth, metastasis, and recurrence. Although recent studies have made great progress towards elucidating the epigenetic regulation of MAGE family, the transcriptional programs controlling their aberrant expression are still not fully understood and much yet is to be discovered. More mechanism studies concerning MAGE function and regulation will provide some new alternative strategies targeting MAGEs in multiple types of cancers.

## Abbreviations

3′UTR: 3′ Untranslated region
AR: Androgen receptor
ATM: Ataxia-telangiectasia-mutated
CG-X: X-link cancer/germline
CNS: Central nervous system
CTAs: Cancer-testis antigens
DNMTs: DNA methyltransferases
EGF: Epidermalgrowth factor
ERα: Estrogen receptor α
FGFR2-IIIb: Fibroblast growth factor receptor 2-IIIb
H3K9me3: Histone 3 lysine 9 trimethylation
KZNFs: KRAB domain zinc finger transcription factors
lncRNAs: Long non-coding RNAs
MAGE: Melanoma Antigens Genes
MBD: Methyl-CpG binding domain
MitA: Mithramycin A
ncRNAs: Non-protein-coding RNAs
PR: Progesterone receptor
PRC-2: Polycomb repressor complex
Rbx1: RING Box protein-1
SKIP: Ski interacting protein
TRD: Transcriptional repression domain
TSA: Histone deacetylases inhibitor trichostatin A
TSS: Transcription start site
